# Not Just Sjögren's: A Rare Life-Threatening Manifestation in a Pediatric Patient

**DOI:** 10.7759/cureus.97434

**Published:** 2025-11-21

**Authors:** Elise Dahan, Abigail Cooley, Kendel Ridgeway, Basil Fathalla, Tarek Husien

**Affiliations:** 1 Pediatrics, Detroit Medical Center (DMC) Children's Hospital of Michigan, Detroit, USA; 2 Rheumatology, Detroit Medical Center (DMC) Children's Hospital of Michigan, Detroit, USA

**Keywords:** autoimmune disease, cytokine storm, hemophagocytic lymphohistiocytosis (hlh), macrophage activation syndrome, sjögren’s syndrome

## Abstract

Macrophage activation syndrome (MAS), a severe hyperinflammatory state within the spectrum of hemophagocytic lymphohistiocytosis (HLH), is a life-threatening complication most commonly associated with rheumatologic disorders such as systemic juvenile idiopathic arthritis. Pediatric Sjögren’s disease, however, is a rare and underrecognized etiology. We present the case of an eight-year-old female patient with no prior medical history who developed MAS in the setting of newly diagnosed Sjögren’s disease, notably without classical sicca symptoms. Her course was marked by progressive systemic inflammation, multiorgan involvement, and laboratory findings suggestive of HLH/MAS. Diagnosis was confirmed through bone marrow biopsy and rheumatologic evaluation, and she responded favorably to immunosuppressive therapy with anakinra and corticosteroids. This case highlights the diagnostic challenges of MAS in atypical pediatric presentations and underscores the need for broader clinical suspicion in cytokine storm syndromes, particularly when established classification criteria are lacking.

## Introduction

Sjögren's disease is a chronic autoimmune disease affecting primarily the lacrimal and salivary glands. Extra-glandular systemic involvement can occur, such as arthritis, interstitial lung disease, and renal disease. Sjögren's disease is common in adults [1]; the exact prevalence and incidence of juvenile-onset disease are unknown [2]. The term primary Sjögren’s disease refers to Sjögren’s disease in the absence of comorbid autoimmune conditions. In contrast, secondary Sjögren’s disease occurs in the context of other systemic connective tissue diseases. Sjögren's disease characteristically presents in adults with sicca manifestations (xerostomia and xerophthalmia); however, these symptoms are scarce in the pediatric population, often leading to delayed diagnosis [2]. A validated classification for adult-onset primary Sjögren’s disease was published in 2016 [3], but there have been no validated classification criteria for the pediatric age group. Juvenile primary Sjögren's disease often presents with recurrent parotitis, non-specific manifestations such as fever, lymphadenopathy, and other rheumatic manifestations such as arthritis [2]. The development of macrophage activation syndrome (MAS) secondary to Sjögren’s disease is rarely reported, even among adults. Within the literature, this is the first case of MAS as a primary presentation of Sjögren's disease in a pediatric patient.

Hemophagocytic lymphohistiocytosis (HLH) represents a medical emergency characterized by an excessive, potentially fatal immune activation that can originate spontaneously (primary) or be triggered by infectious, oncologic, or rheumatologic disorders (secondary). MAS is a subset of secondary HLH triggered by autoimmune conditions that manifests as a severe inflammatory response known as a cytokine storm, often progressing to multisystem organ failure (multiple organ dysfunction syndrome (MODS)). Diagnosis is based on persistent fevers, hyperferritinemia, transaminitis, cytopenia, coagulopathy resembling disseminated intravascular coagulation (DIC), and neurologic sequela. The development of MAS secondary to systemic juvenile idiopathic arthritis (sJIA), Kawasaki disease, and systemic lupus erythematosus (SLE) has been previously reported [4]. Most clinical data describe MAS as a complication of sJIA, with approximately 10% of patients developing fulminant MAS. Subclinical MAS may occur in 30% to 40% of children with confirmed or suspected sJIA [5]. Current pediatric diagnostic criteria are only established in the setting of sJIA in the 2016 MAS criteria, which include persistent fever, ferritin > 684 ng/mL, and at least two of the following: platelet counts < 181 K/cumm, aspartate aminotransferase (AST) > 48 U/L, triglycerides > 1.76 mmol/L, and fibrinogen < 10.6 µmol/L [6]. However, juvenile Sjögren's disease is rarely documented and currently lacks a validated classification criterion in the pediatric population. The development of MAS secondary to Sjögren’s disease is infrequently reported, even among adults, which may be attributed to the lack of established diagnostic criteria or late recognition due to its potential confusion with an acute flare of the underlying condition [7].

This article was previously presented as a poster at the 2024 AAP National Conference & Exhibition on September 28, 2024.

## Case presentation

This is an eight-year-old female patient with no past medical history who presented to the emergency department with a seven-day history of fever, sore throat, generalized myalgias, and arthralgias. Two weeks prior to admission, she was seen at her primary care office for complaints of a cough and was diagnosed with an upper respiratory infection. Due to continuing symptoms, she was seen by her pediatrician a second time one day prior to presentation and diagnosed with streptococcal pharyngitis and prescribed a 10-day course of amoxicillin-clavulanate. Following one dose of the second antibiotic course, arthralgias intensified to the point where she was unable to ambulate, prompting her presentation to the emergency department.

Initial inpatient evaluation revealed coagulopathy, transaminitis, worsening pain, and refusal to ambulate, prompting a broadening of the differential beyond acute pharyngitis. Upon admission to the inpatient unit, despite negative blood cultures, she continued to have persistent fevers, and the infectious disease team recommended intravenous (IV) ampicillin-sulbactam. Initially, vital signs were notable for high intermittent fevers (T-max 40.0°C), tachycardia (heart rate (HR) 124-166 bpm), and tachypnea (respiratory rate 24-28), with adequate oxygen saturation on room air. Physical examination was significant for cracked lips and conjunctival injection without annular sparing; however, the exam was negative for cervical lymphadenopathy, respiratory findings, extremity changes, dry eyes, or dry oral mucosa. The joint exam was unremarkable, without effusion, and the patient had a normal range of motion.

Laboratory studies on hospital day one were significant for elevated inflammatory markers, transaminitis, and hypoalbuminemia. The respiratory pathogen profile, Epstein-Barr virus (EBV), and cytomegalovirus (CMV) were all negative. COVID PCR was negative, but COVID IgG antibody was found to be elevated at 4.26 (reference <0.81 normal), indicative of past infection. Initial presentation was suggestive of multisystem inflammatory syndrome in children (MIS-C) versus incomplete Kawasaki disease. Kawasaki disease was considered less likely due to the patient being outside of the normal age range and meeting only two physical exam criteria with downtrending platelets and a normal echocardiogram. Eighty grams of IV immunoglobulins (IVIG) was administered on day three of admission for suspected MIS-C.

**Figure 1:**
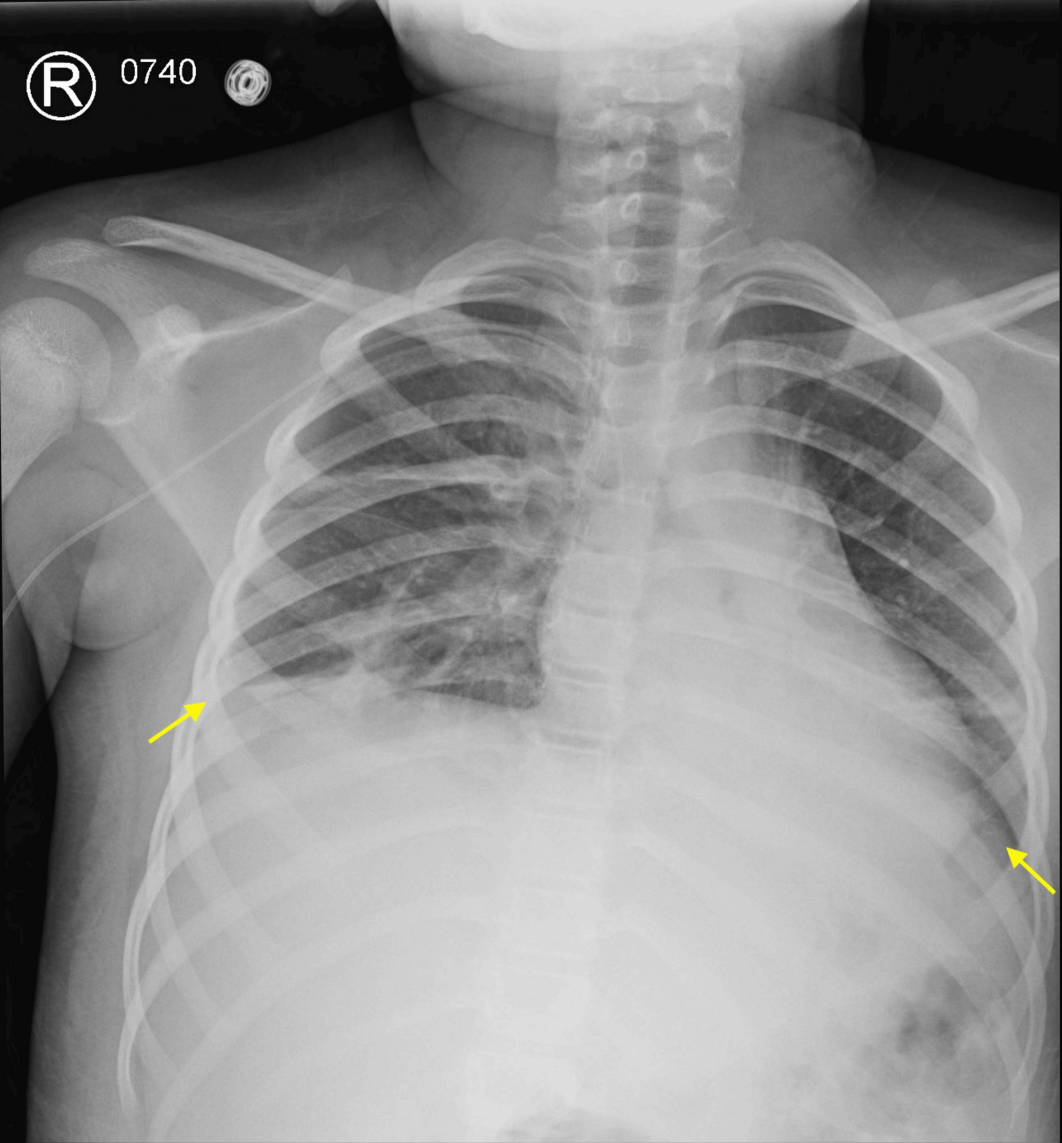
Chest x-ray demonstrating bilateral pleural effusions

Despite IVIG, there was no clinical improvement, continued up-trending inflammatory markers, and worsening transaminitis (alanine aminotransferase (ALT) 141-264 U/L, AST 637-1,363 U/L; Ref: AST 11-52 U/L, AST 13-39 U/L) (Table 1). On day four, the patient was noted to have elevated troponin and significant hyperferritinemia (maximum ferritin 28,650 ng/mL; Ref: 11-308.6 ng/mL). She also developed mucocutaneous symptoms including epistaxis, gingival bleeding, and hemoptysis with multiple blood clots concerning for pulmonary hemorrhage. The patient was found to be coagulopathic on hospital day six. Laboratory evaluation at that time revealed downtrending hemoglobin from 10.3 to 8.1 g/dL (reference range: 11.6-15.1 g/dL) and thrombocytopenia to 114 K/cumm (reference range: 130-450 K/cumm). This prompted the patient to receive one unit of each fresh-frozen plasma and packed red blood cells, followed by 20 mg of furosemide. On hospital day seven, the patient began to have worsening desaturation and tachypnea requiring a 28% Ventimask. She continued to have respiratory decompensation with the ultimate requirement of a high-flow nasal cannula at 20 L 100% FiO_2_. Chest x-ray demonstrated bilateral pleural effusions (Figure 1).

**Table 1:** Summary of pertinent laboratory results ALT: alanine aminotransferase; AST: aspartate aminotransferase; CPK: creatine phosphokinase; LDH: lactate dehydrogenase; RBC: red blood cell; PT: prothrombin time; PTT: partial thromboplastin time; INR: international normalized ratio

Lab tests	Initial values	Reference ranges
CRP	20.1	<5 mg/L
ALT	264	7-52 U/L
AST	1,363	13-39 U/L
CPK	11,799	30-223 U/L
LDH	>6,000	140-271 U/L
Triglycerides	245	<150 mg/dL
Ferritin	28,650	11-306.8 ng/mL
Troponin	151	3-17 ng/L
Total protein	5.1	6.2-7.4 g/dL
Albumin	2.3	3.9-4.9 g/dL
WBC	42.9	4.1-11.3 K/cumm
RBC	2.82	4.23-5.43 M/cumm
Hemoglobin	6.7	11.6-15.1 g/dL
Platelets	42	130-450 K/cumm
PT	18.4	9.4-11.7 s
PTT	56.9	23.1-33.1 s
INR	1.79	0.87-1.1
COVID PCR	Not detected	N/A
COVID IgG antibody	4.26	<0.81 S/CO

Rheumatology and hematology services were consulted to investigate the etiology of her condition, given her significant inflammatory response. At the time of the initial hematology consultation on day six, the patient met three out of eight criteria for HLH based on the guidelines detailed below, with a temperature of >38.0°C, hypofibrinogenemia at 64 mg/dL, and hyperferritinemia at 17,400 ng/mL. A bone marrow biopsy was performed on day seven, which revealed hemophagocytosis of the marrow, indicating HLH vs. MAS. By day 10 of admission, platelets had decreased to 88 x 10^9^/L, and she was known to have a bone marrow biopsy showing hemophagocytosis, meeting the five required criteria for the diagnosis of HLH.

A diagnosis of HLH can be made either by identifying a causative molecular mutation or by meeting at least five of the following eight clinical criteria: persistent fever, enlarged spleen, cytopenias affecting at least two blood cell lines (hemoglobin < 9 g/dL or <10 g/dL in newborns; platelets < 100 × 10^9^/L; neutrophils < 1.0 × 10^9^/L), elevated triglycerides (>265 mg/dL) or low fibrinogen (<150 mg/dL), markedly increased ferritin levels (>500 ng/mL), elevated soluble CD25 (>2,400 U/mL), evidence of hemophagocytosis on tissue biopsy, and reduced or absent natural killer (NK) cell activity [8].

**Figure 2:**
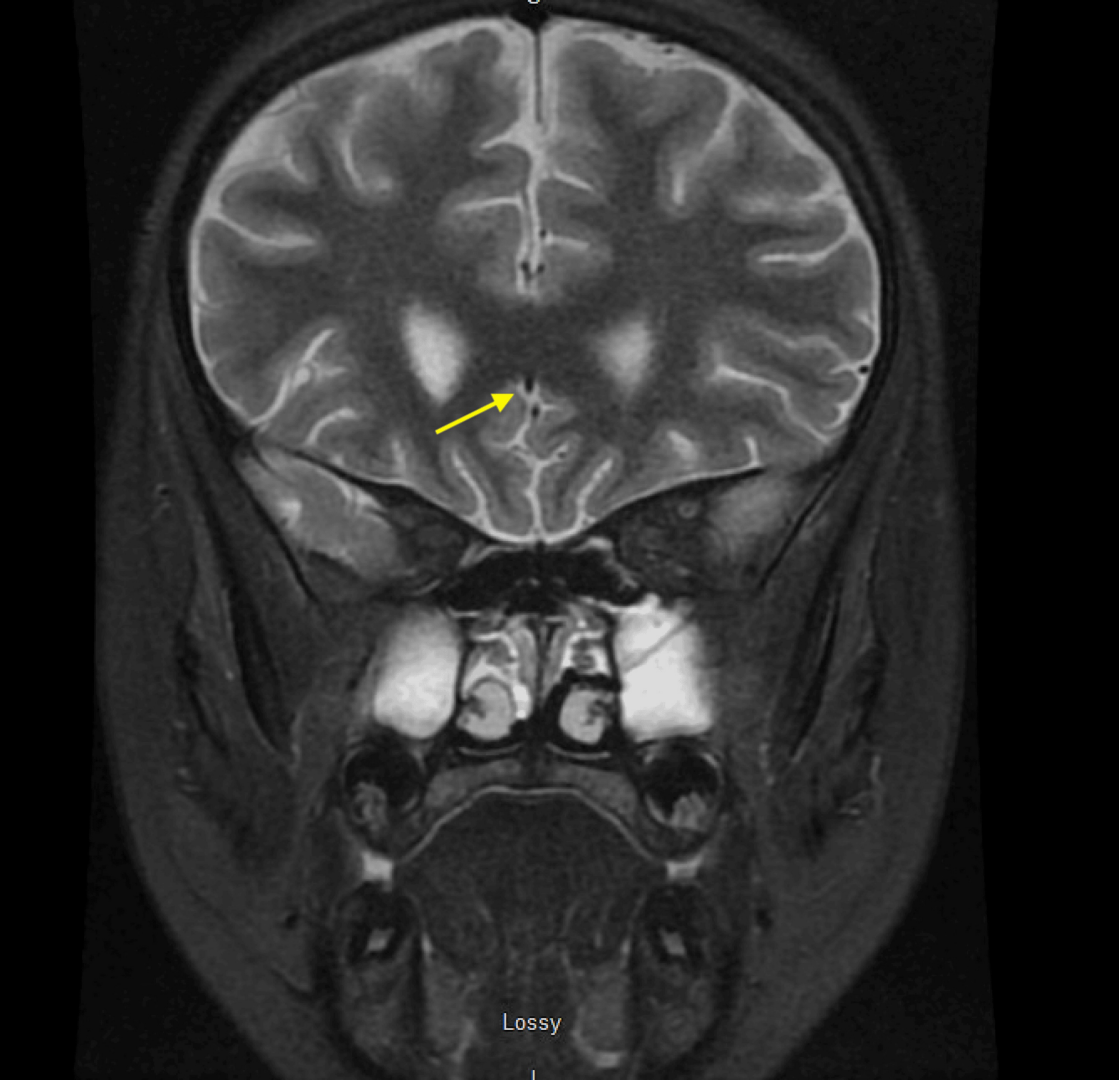
MRI brain w/w/o contrast showing punctate microhemorrhages as well as supra- and infratentorial volume loss MRI: magnetic resonance imaging

**Figure 3:**
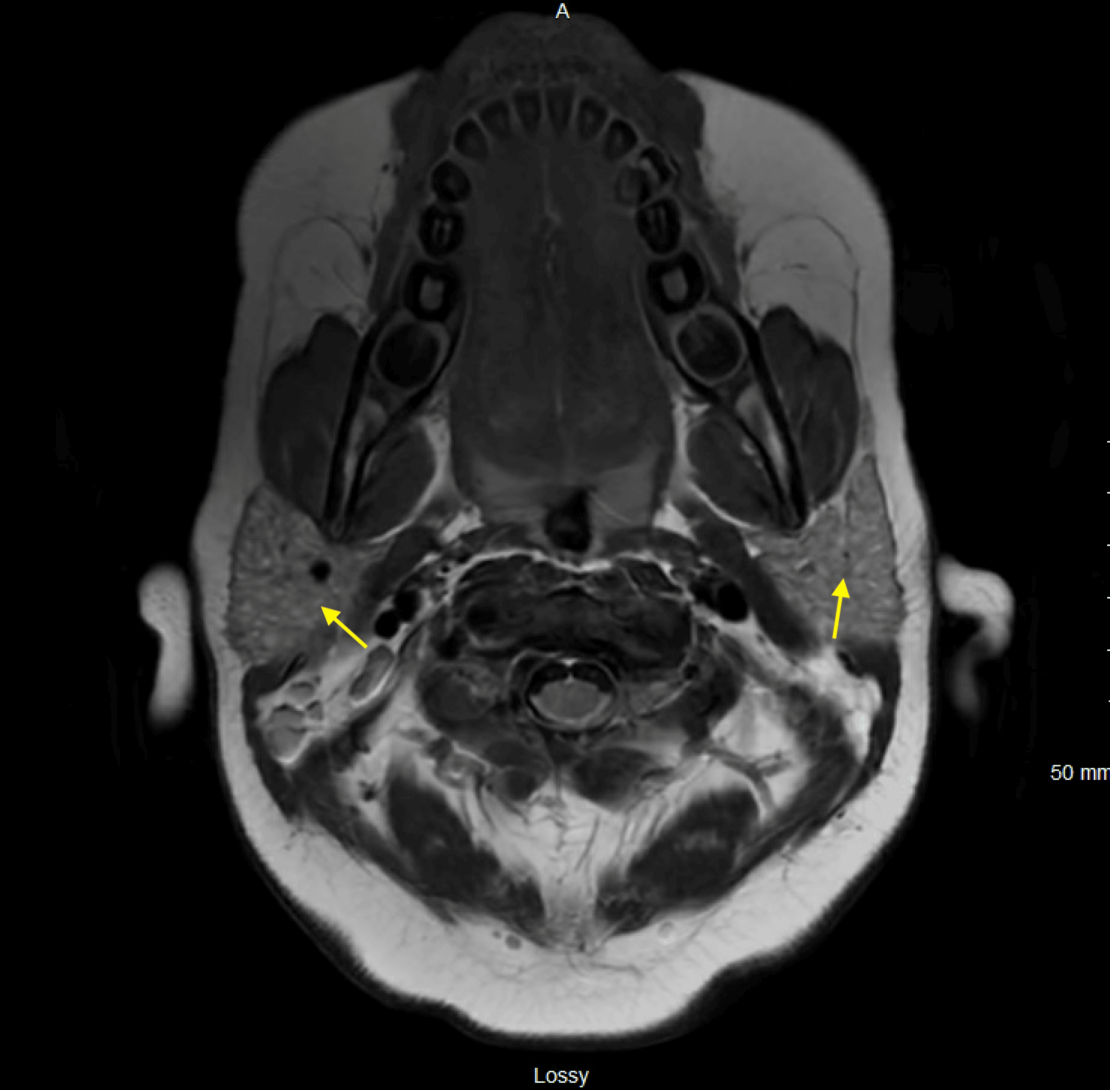
MRI brain w/w/o contrast showing multiple tiny cystic lesions in the bilateral parotid glands suggestive of Sjögren's disease MRI: magnetic resonance imaging

After the biopsy results, treatment was escalated to anakinra 100 mg every six hours, as well as pulse doses of IV methylprednisolone 1,000 mg daily for three days, followed by a maintenance dose of 60 mg daily until discharge. Treatment for MAS started with anakinra and steroids even before confirming the triggering etiology, as MAS is life-threatening and treatment with steroids and anakinra is standard in such cases [9,10], in addition to treating the underlying etiology once identified. On day eight, IV ampicillin-sulbactam was changed to IV cefepime and vancomycin to treat for an additional seven days, as infectious causes had not yet been ruled out and the patient was critically ill; however, all cultures remained negative. Magnetic resonance imaging (MRI) with angiography (MRA) and venography (MRV) was done for new-onset headaches on day 17, demonstrating infra- and supratentorial volume loss, likely due to steroid treatment and petechial hemorrhages in the pons, likely secondary to coagulopathy (Figure 2). An incidental finding was sialadenitis of the bilateral parotid glands (Figure 3), which prompted the inclusion of Sjögren's disease in our differential, especially in view of the detected anti-SSA antibodies done as part of the work-up for SLE.

Additional work-up for MAS triggering rheumatologic diseases revealed a diagnosis of Sjögren's disease, including a positive antinuclear antibody (ANA) at 1:160, anti-SSA antibodies at 55.8 AAU/mL (Ref: 0-24.9 AAU/mL), and multiple cystic lesions within the bilateral parotid glands on MRI. MIS-C and Kawasaki disease were both considered as the etiology of her symptoms; however, severe clinical decompensation and worsening laboratory evaluation despite receiving treatment for these conditions in addition to her positive rheumatic work-up revealed MAS as a more likely etiology. Potential infectious triggers had been treated with multiple courses of broad-spectrum antibiotics. The presentation was considered more consistent with MAS, as the work-up for malignancy, including a bone marrow biopsy and peripheral smear, was negative. A congenital HLH panel was negative for any genetic mutations known to cause primary HLH.

She was continued on a regimen of anakinra and methylprednisolone with significant improvement in symptoms and laboratory studies. The patient’s fever, tachycardia, and respiratory status gradually improved, allowing for successful weaning from oxygen support. However, due to her prolonged immobility and a 10-day stay in the pediatric intensive care unit, she required physical therapy to address her inability to ambulate. She was safely transferred back to the pediatric ward for continued medical management and later transitioned to the inpatient rehabilitation unit. She was discharged on day 23 of admission with a home regimen of 100 mg anakinra daily and 60 mg of oral prednisolone daily, which were slowly weaned in the outpatient setting based on frequent laboratory studies. An outpatient salivary gland biopsy was completed after the resolution of acute illness. Results confirmed parotitis with nodular lymphocytic aggregates and focal infiltration of lymphocytes into the ductular epithelium consistent with Sjögren's disease. Three months following admission, she was transitioned to maintenance therapy for primary Sjögren's with mycophenolate mofetil and hydroxychloroquine. Nephrology also followed her for steroid-induced hypertension, which resolved after discontinuation of steroids. At the time of this writing, 18 months following discharge, the patient has not had any relapse of MAS since she was discharged and has continued treatment of her underlying Sjögren's disease.

## Discussion

Macrophages help maintain control of the inflammatory process through mediated immune defense. Monocytes are mobile macrophages that circulate in the bloodstream and migrate into tissues, where they differentiate into histiocytes and carry out phagocytosis of antigens. This migration is driven by lymphokines such as interferon-gamma (IFN-γ) and tumor necrosis factor-alpha (TNF-α). T helper 1 (Th1) lymphocytes can continuously produce IFN-γ, supporting ongoing macrophage activation. The interaction between macrophages and Th1 cells is critical for the development of cell-mediated immunity. Activated macrophages secrete proinflammatory cytokines that are vital for host defense but can cause significant tissue damage if the inflammatory response is not properly regulated. In MAS, there is an overproliferation of Th1 lymphocytes, resulting in excessive and aberrant macrophage activation and proliferation [11]. A key laboratory feature of MAS is hyperferritinemia, and activated macrophages are a major contributor to elevated serum ferritin levels in the acute inflammatory phase [7].

Although precise etiology remains uncertain, an increased frequency of heterozygous mutations in familial HLH-related genes has been identified among MAS patients. This genetic overlap between HLH and MAS offers potential insight into similar underlying mechanisms. Heterozygous mutations in familial HLH-associated genes (such as PRF1, LYST, RAB27A, UNC13D, STXBP2, and STX11) are detected in up to 40% of MAS cases, which is notably higher than the 15% prevalence reported in the general population and among disease control groups. These familial mutations impair the perforin-dependent cytolytic pathway, which prevents cytotoxic lymphocytes from effectively destroying mutated antigen-presenting cells, resulting in excessive release of pro-inflammatory cytokines that drive clinical manifestations of MAS [5]. 

In a systematic review examining MAS in rheumatic patients, only three out of 421 cases were associated with Sjögren's disease [12]. Batu et al. present a similar case [13], which they reported as the first identified case of pediatric MAS secondary to Sjögren’s disease. Though the initial presentation was similar to our case with arthralgias, the cited patient also had classic sicca symptoms of Sjögren’s, including xerophthalmia and xerostomia. Additionally, the patient cited had an established diagnosis of Sjögren's years prior to the onset of MAS - our case is the only case found on our search of Sjögren's presenting as MAS. Pediatric Sjögren's is an uncommon primary disease presentation, decreasing in prevalence despite the rise in total global burden of autoimmunity [2].

Unfortunately, MAS often remains underrecognized and poorly defined until later stages of disease [5], with diagnosis made even more difficult in pediatric patients with no known rheumatologic history, requiring a high index of clinical suspicion in critically ill patients. Differentials for MAS include other hyperinflammatory states such as MODS, septic shock, DIC, and flares of a primary rheumatologic condition [4]. Current diagnostic criteria for Sjögren’s disease and MAS in the pediatric population continue to be lacking, and further work is needed to adequately define these severe syndromes with the goal of early identification and treatment.

Identifying the underlying etiology of cytokine storm may prove to be time-consuming or even impossible (in cases of idiopathic HLH/MAS), resulting in delayed treatment and increased morbidity. Given the importance of prompt treatment, the use of cytokine-targeted drugs has shifted the management of MAS from identifying and treating the underlying etiology to promptly treating the inflammatory response. Anakinra, an IL-1 receptor antagonist, is a recombinant protein that prevents IL-1 binding at its receptor site and activating downstream pro-inflammatory signaling pathways [5]. A retrospective study done at Children’s of Alabama showed that when initiated within the first five days of admission, anakinra significantly decreased mortality in patients with non-malignancy-associated MAS [14].

Sjögren’s disease is a lesser-known etiology of MAS in the pediatric population. Clinicians should consider Sjögren's disease in pediatric patients with unexplained hyperferritinemia and multiorgan inflammation, even without sicca symptoms, and should include salivary gland imaging in the evaluation of potential MAS. Anakinra and steroids were an effective treatment in this case. The patient achieved recovery after initiation of anakinra and steroids and has avoided relapse of MAS by continuing treatment for Sjögren’s disease in the outpatient setting. Long-term untreated Sjögren’s disease is complicated by increased morbidity due to worsening sicca symptoms, fatigue, pain, and an increased risk of malignancies such as non-Hodgkin and thyroid cancer [15].

## Conclusions

MAS in the context of Sjögren's disease is scarcely reported. Prompt recognition and treatment of life-threatening MAS is crucial, with delay in treatment associated with rapid mortality. The absence of sicca manifestations and other features of Sjögren’s disease and SLE in patients presenting with new-onset MAS should not deter the clinician from investigating these entities as possible triggers of MAS, underscoring the need for clinicians to maintain a broad differential diagnosis when faced with systemic inflammatory signs and features of cytokine storm.
